# Clinical efficacy and safety of acupuncture combined with statin in dyslipidemia: A meta-analysis and system review

**DOI:** 10.1097/MD.0000000000039663

**Published:** 2024-09-13

**Authors:** Xinyu Liu, Kun Chen, Fujian Chen

**Affiliations:** a Department of Science and Education, Foshan Shunde District Traditional Chinese Medicine Hospital: Guangzhou University of Traditional Chinese Medicine ShunDe Traditional Chinese Medicine Hospital, FoShan, China; b Clinical Medical College of Acupuncture Moxibustion, Guangzhou University of Chinese Medicine (Guangzhou University of Chinese Medicine Equivalent Mechanics Applicants for Master’s Degree), Guangzhou, China; c Department of Preventive Treatment of Disease, Foshan Shunde District Traditional Chinese Medicine Hospital: Guangzhou University of Traditional Chinese Medicine, FoShan, China.

**Keywords:** acupuncture, dyslipidemia, meta-analysis, statin

## Abstract

**Background:**

This study aims to systematically evaluate the clinical efficacy and safety of acupuncture in combination with statin therapy compared to statin monotherapy for the treatment of dyslipidemia.

**Methods:**

A comprehensive search for relevant randomized controlled trials assessing the clinical efficacy of acupuncture and statin combination in the treatment of dyslipidemia was conducted. Databases including PubMed, EMbase, Web of Science, Cochrane Library, China National Knowledge Infrastructure, China Biology Medicine disc, Wanfang database, and China Science and Technology Journal Database were searched up to October 27, 2023.

**Results:**

Sixteen Chinese-language studies involving a total of 1333 subjects were included for analysis. The meta-analysis revealed that the total effective rate of acupuncture combined with statin was significantly higher than that of statin alone (odds ratios = 3.32, 95% confidence intervals [CI] = 2.33 to 4.72). Furthermore, the combination of acupuncture with statin treatment resulted in a significant reduction in triglyceride levels (mean differences [MD] = −0.72 mmol/L, 95% CI = −1.05 to −0.4), total cholesterol levels (MD = −0.79 mmol/L, 95% CI = −1.07 to −0.51), low-density lipoprotein cholesterol levels (MD = −0.61 mmol/L, 95% CI = −0.95 to −0.27) and traditional Chinese medicine syndrome integral (MD = −1.32, 95% CI = −1.75 to −0.89), compared to statin treatment alone. Additionally, the high-density lipoprotein cholesterol level was higher in the combined acupuncture and statin treatment group than in the statin treatment alone group (MD = 0.44 mmol/L, 95% CI = 0.09 to 0.79). Notably, the rate of adverse reactions reported with combined acupuncture and statin treatment was lower than that with statin therapy alone.

**Conclusion:**

Our findings support the potential of acupuncture combined with statin as a viable clinical treatment option for dyslipidemia. However, it is important to note that current research on the mechanism of acupuncture for lipid-lowering has not yielded definitive results, and there are inherent biases in the conducted clinical studies.

## 1. Introduction

Dyslipidemia, characterized by elevated triglycerides (TG), total cholesterol (TC), low-density lipoprotein cholesterol (LDL-C), and reduced high-density lipoprotein cholesterol (HDL-C), is a significant risk factor for atherosclerotic cardiovascular disease (ASCVD), presenting a substantial challenge in developing countries.^[1]^ Based on a recent study published in the journal Nature,^[2]^ it has been observed that developing countries in East Asia, Southeast Asia, and Oceania have witnessed a substantial increase in non-HDL-C levels over the past 4 decades, with the highest surge recorded at 0.88 mmol/L. Notably, China has demonstrated one of the most rapid escalations in non-HDL-C levels globally, with the age-standardized average non-HDL-C level for Chinese men rising from 2.77 mmol/L in 1980 to 3.38 mmol/L in 2018.^[2]^ These trends have also led to a significant global burden, with approximately 3.9 million people dying from hypercholesterolemia each year, half of whom are in China and other Southeast Asian countries.^[2]^ While health care policies and targeted LDL-C lowering drugs have shown promise in reducing ASCVD burden, challenges persist.^[3]^ Statins, the primary medication for lipid regulation, have demonstrated significant clinical lipid-lowering effects. However, issues such as statin intolerance, failure to achieve desired lipid-lowering effects at moderate intensity, and an increased risk of new-onset diabetes at high intensity remain prevalent. Although new lipid-lowering drugs like proprotein convertase subtilisin/kexin type 9 inhibitors offer additional lipid adjustment potential, their high cost impedes widespread adoption in developing countries. Acupuncture, as a traditional Chinese medicine (TCM) treatment, has garnered attention for its clinical efficacy in recent years. It has been utilized either independently or in combination with statin for dyslipidemia treatment in TCM institutions. Therefore, this meta-analysis aims to provide evidence-based insights into the efficacy and safety of acupuncture combined with statin in managing dyslipidemia.

## 2. Research design and methods

### 2.1. Inclusion and exclusion criteria

Participant inclusion criteria: Our study does not impose restrictions based on age, gender, or source of cases for participants. However, participants must have been diagnosed with primary dyslipidemia in accordance with the Chinese guidelines.^[4]^ Exclusion criteria encompass individuals with other serious diseases or complications, as well as those with unstable vital signs. Pregnant women are also ineligible for participation. Intervention: The study aimed to compare the effects of statin therapy alone with those of combined statin and acupuncture treatment. The control group received primarily statin therapy, while the intervention group underwent acupuncture therapy, including electroacupuncture, manual acupuncture, auricular acupuncture, and warm acupuncture, in conjunction with an equivalent statin dosage. Acupoint injection and acupoint embedding were excluded to ensure the specific intervention effect of acupuncture combined with statin. Outcome Measures: The primary outcome measure focused on the total effective rate, as per the Clinical Guiding Principles of New Chinese Medicine.^[5]^ Secondary outcomes encompassed serum lipids levels, adverse reactions, and TCM syndrome integral, with standards based on Clinical Guiding Principles of New Chinese Medicine.^[5]^ Study Design: The eligibility criteria were defined as randomized controlled trials (RCTs) employing a double-blind, single-blind, or non-blind design. Trials were excluded based on the following criteria: duplicate publication, unavailability of full-text access, and inability to obtain test data.

### 2.2. Search strategy

An intensive electronic search from their inception through October 27, 2023 was conducted using Embase, PubMed, Cochrane Library, Web of Science, China National Knowledge Infrastructure, Wan Fang database, China Biology Medicine disc, China Science and Technology Journal Database. Similarly, Searched the clinical trial registration platform: Chinese Clinical Trial Registey, U.S.National Library of Medicine ClinicalTrials.gov. Our search terms include: Dyslipidemia, Dyslipoproteinemias, Dyslipoproteinemia, Hyperlipemia, Hyperlipemias, Hyperlipidemia, Lipidemia, Lipidemias Lipemia, Lipemias, Acupuncture Therapy, Acupuncture Treatment, Acupuncture Treatments, Treatment, Acupuncture, Therapy, Acupuncture, Pharmacoacupuncture Treatment, Treatment, Pharmacoacupuncture, Pharmacoacupuncture Therapy, Therapy, Pharmacoacupuncture, Acupotomy, Acupotomies, Acupuncture, Pharmacopuncture, Randomized controlled trial, Controlled clinical trial, Randomized, Controlled, Trial, Random, Groups.

### 2.3. Data selection and extraction

The initial literature screening was independently conducted by 2 researchers (X.L. and K.C.). The process involved an initial assessment based on the title and abstract to determine potential exclusions. Subsequently, the full text of the articles was reviewed to confirm exclusions. In cases of disagreement between the 2 evaluators, a reevaluation of the full text was performed. If discrepancies persisted, a third evaluator (F.C.) made the final decision on inclusion. Information extracted from the literature included the title, first author, publication year, sample size, participant characteristics, disease duration, random sequence generation, randomization concealment, blinding, intervention duration, specific intervention, outcome indicators, and other relevant details. Detailed data were then entered into a pre-designed data extraction table and managed using Word and Excel.

### 2.4. Quality assessment

Once a decision was made regarding the inclusion of literature in the analysis, full articles were assessed using the Cochrane Risk of Bias tool.^[6]^ In cases of disagreement, efforts were made to reach a consensus, and if necessary, a third party (F.C.) was consulted. The Cochrane Risk of Bias tool comprises 6 individual domains, including: random sequence generation (selection bias), allocation concealment (selection bias), blinding of participants and personnel (performance bias), blinding of outcome assessment (detection bias), incomplete outcome data (attrition bias), selective reporting (reporting bias), and other bias. Each domain was categorized as presenting a low risk of bias, high risk of bias, or unclear risk of bias.

### 2.5. Statistical analyses

All data analysis in this study was conducted using R version 4.1.2 (https://www.r-project.org/) software. Binary outcomes were summarized using odds ratios with 95% confidence intervals (CI), while continuous outcomes were presented as weighted mean differences (MD) with 95% CI. Heterogeneity was evaluated using the chi-square test and the Higgins *I*^2^ test. Publication bias was assessed using funnel plots, Begg’s test, and Egger’s test, calculated with R software. To address potential outcome heterogeneity, sensitivity analyses were performed through subgroup analyses based on mean age, disease duration, and intervention duration, as well as through case-by-case exclusions.

### 2.6. Ethics approval/institutional review board

This type of study is a meta-analysis, so it is exempt from ethical approval.

## 3. Results

### 3.1. Search results

**Figure 1.**
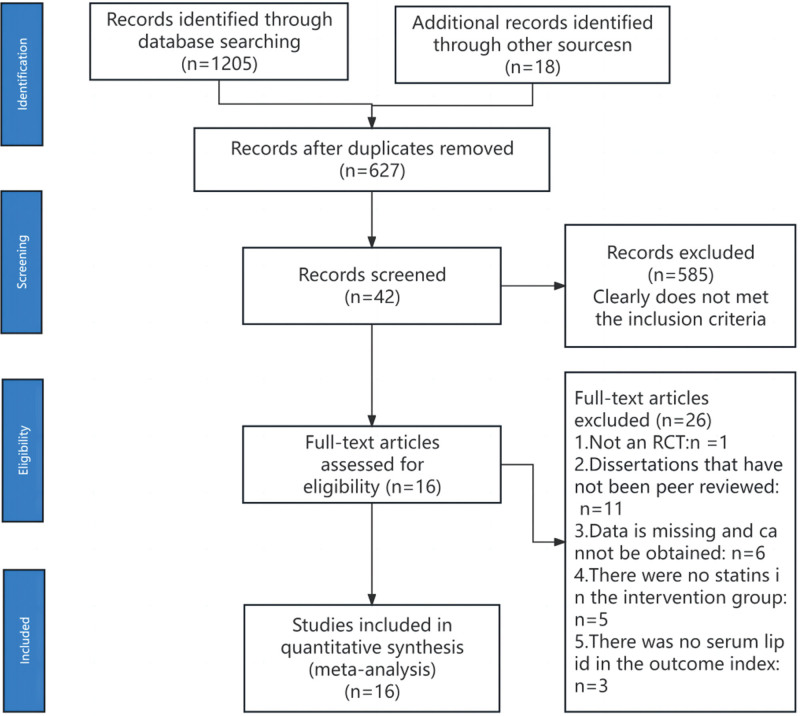
PRISMA diagram of the search strategy. PRISMA = Preferred Reporting Items for Systematic Reviews and Meta-Analyses.

The initial search strategy identified 1205 studies, with no clinical registration trials and 1205 published articles (PubMed: 38, Embase: 52, Cochrane Library: 60, Web of Science: 26, China Biology Medicine disc: 154, China National Knowledge Infrastructure: 433, Wan Fang database: 220, China Science and Technology Journal Database: 222). After manually reviewing the literature, 596 duplicate articles were excluded. Secondly, 567 articles were excluded by reading the title and abstract. Subsequently, after full-text review, 21 articles were further excluded for reasons such as non-RCT study design, absence of a statin control group, missing or unavailable data, lack of peer review, or absence of serum lipid outcome data. Ultimately, 16 articles were included in the final analysis. A diagram illustrating the search strategy, following the Preferred Reporting Items for Systematic Reviews and Meta-Analyses guidelines,^[7]^ is available in Figure 1.

### 3.2. Characteristics of included studies

Selected characteristics of the included studies are reported in Table 1.

**Table 1:** Characteristics of included studies.

Study	Participants (n)		Mean age	Duration of the disease	Average length of treatment	Intervention		Outcome measures
	Intervention group	Control group				Intervention group	Control group	
Zhiming 2016^[8]^	50	50	49.1	10 d–16 mo	8 wk	Lovastatin + Acupuncture	Lovastatin	Total effective rate, serum lipids level
Chen 2016^[9]^	49	49	57.35	Average 7.1 yr	15 wk	Atorvastatin + Acupuncture	Atorvastatin	Serum lipids level, adverse reactions
Quan 2010^[10]^	46	45		–	4 wk	Fluvastatin + Acupuncture	Fluvastatin	Total effective rate, serum lipids level, adverse reactions, TCM syndrome integral
Ting 2016^[11]^	20	20	55.36	–	1 mo	Atorvastatin + Acupuncture	Atorvastatin	Total effective rate, serum lipids level
Gu 2015^[12]^	30	30		–	3 mo	Atorvastatin + Acupuncture	Atorvastatin	Total effective rate, serum lipids level, adverse reactions
Zhen 2019^[13]^	30	30	63	Average 107 d	2 wk	Rosuvastatin + Acupuncture	Rosuvastatin	Total effective rate, serum lipids level, adverse reactions
Heng 2020^[14]^	45	45	59	Average 7.96 yr	8 wk	Atorvastatin + Acupuncture	Atorvastatin	Total effective rate, serum lipids level
Tang 2014^[15]^	63	57	43.95	Average 5.7 yr	6 wk	Atorvastatin + Acupuncture	Atorvastatin	Total effective rate, serum lipids level, adverse reactions
Sun 2015^[16]^	30	30	50.5	Average 2.85 yr	6 wk	Atorvastatin + Acupuncture	Atorvastatin	Total effective rate, serum lipids level
Shuhong 2023^[17]^	48	48	64.5	Average 35.1 mo	4 mo	Simvastatin + warm acupuncture therapy + herbal teas	Simvastatin	Serum lipids level, TCM syndrome integral, adverse reactions
Liyou 2020^[18]^	35	35	56.36	Average 8.54 yr	4 wk	Simvastatin + Acupuncture + He Wei Jiangzhuo prescription	Simvastatin	Total effective rate, serum lipids level, adverse reactions
Wang 2020^[19]^	50	50	50.03	–	1 mo	Simvastatin + Acupuncture + Aconite Center-Rectifying Decoction	Simvastatin	Total effective rate, serum lipids level, adverse reactions
Ge 2019^[20]^	42	42	61.95	–	16 wk	Simvastatin + Acupuncture + herbal teas	Simvastatin	Serum lipids level
You 2019^[21]^	27	27		–	16 wk	Simvastatin + warm acupuncture therapy + herbal teas	Simvastatin	Serum lipids level, TCM syndrome integral
Zhu 2017^[22]^	50	50	63.5	–	4 mo	Simvastatin + warm acupuncture therapy + herbal teas	Simvastatin	Serum lipids level, TCM syndrome integral
Qi 2015^[23]^	55	55	63.7	–	16 wk	Simvastatin + warm acupuncture + herbal teas	Simvastatin	Total effective rate, serum lipids level, TCM syndrome integral

### 3.3. Quality assessment

**Figure 2.**
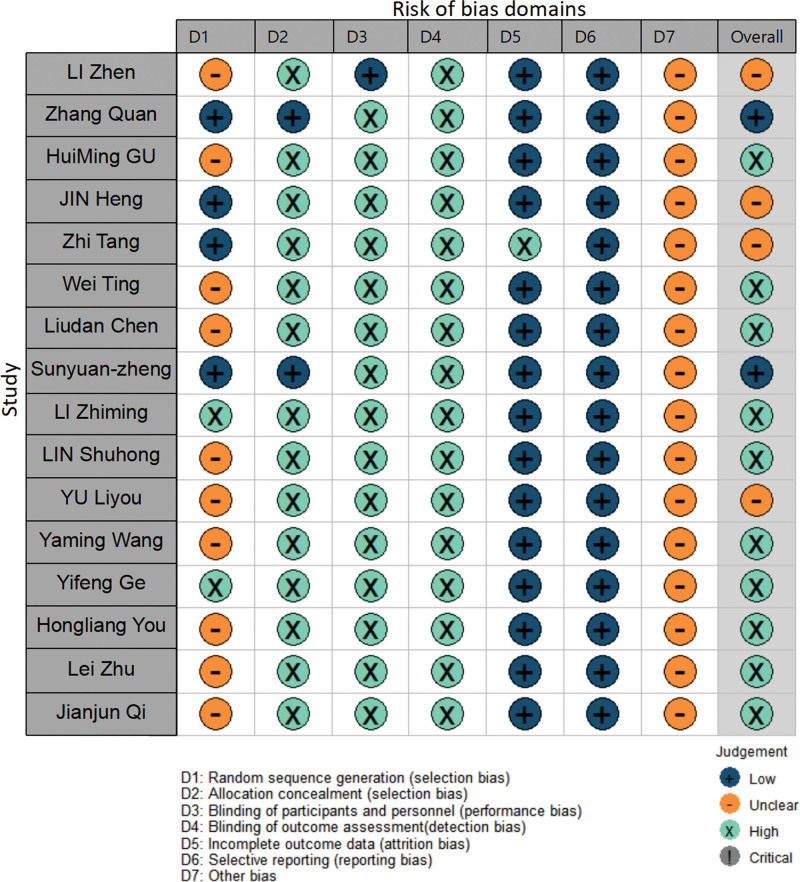
Risk of bias summary.

We evaluated the methodological quality of the included literature according to the Cochrane handbook.^[6]^ No selective reports were found in the 16 included RCTS. There were 15 studies that reported complete results, 14 of which used randomized methods, but the details were unclear; Only 4 of them specifically described the generation of random sequences, 1 study implemented the single-blind method, 2 studies demonstrated allocation concealment, and 16 other biases were not clear. See Figure 2 for details.

### 3.4. Therapeutic evaluation

#### 3.4.1. Total effective rate

**Figure 3.**
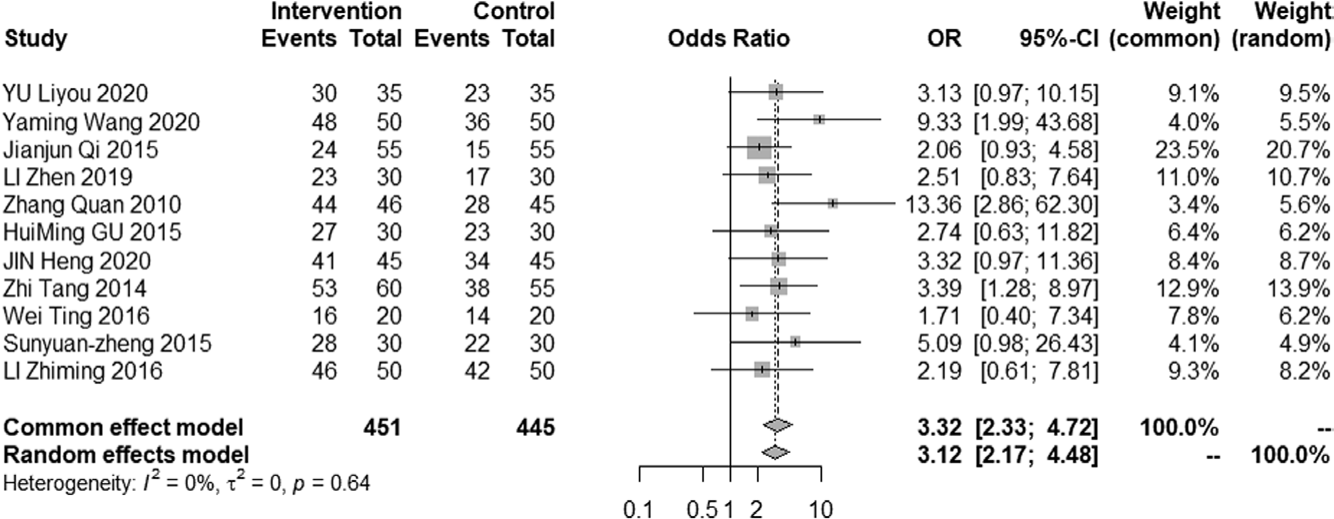
Meta analysis of the total effective rate.

Eleven studies (n = 896) reported the total effective rate for acupuncture combined with statin in the treatment of dyslipidemia. The heterogeneity test results indicated homogeneity among the studies (*P* = .64, *I*^2^ = 0%), and therefore, the fixed-effect model was utilized (Fig. 3). The pooled odds ratio value was 3.12, with a 95% CI of 2.17 to 4.48, demonstrating that acupuncture combined with statin was more effective than statin alone.

#### 3.4.2. TG level

**Figure 4.**
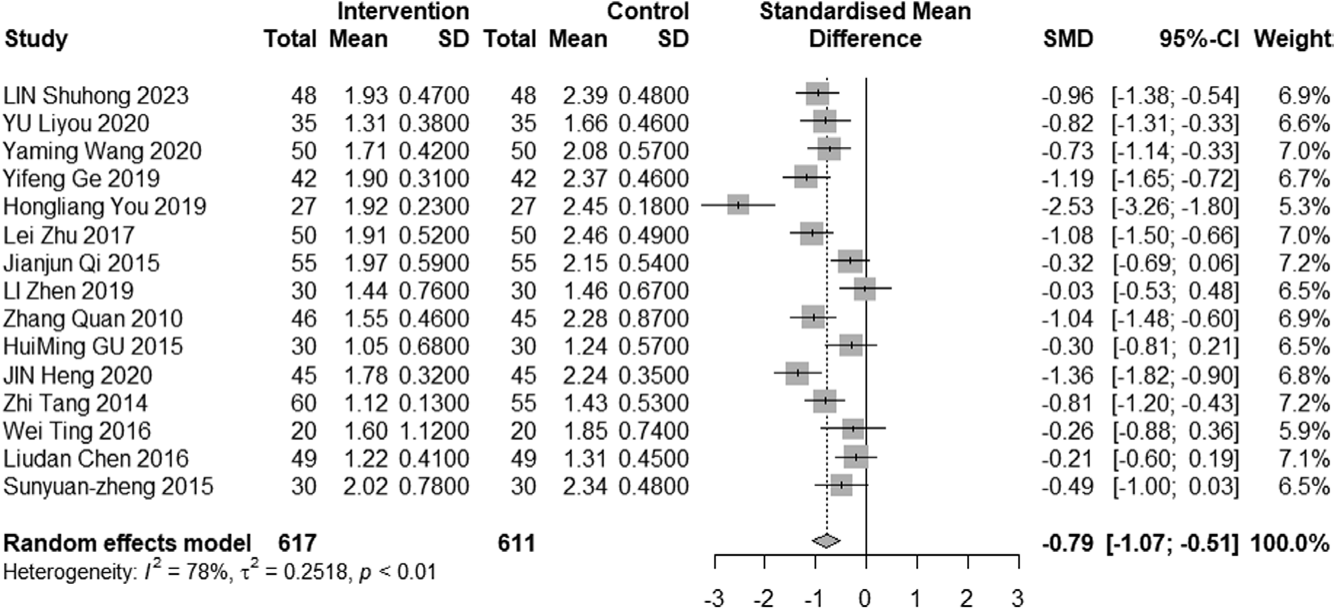
Meta analysis of TG level. TG = triglyceride.

**Figure 5.**
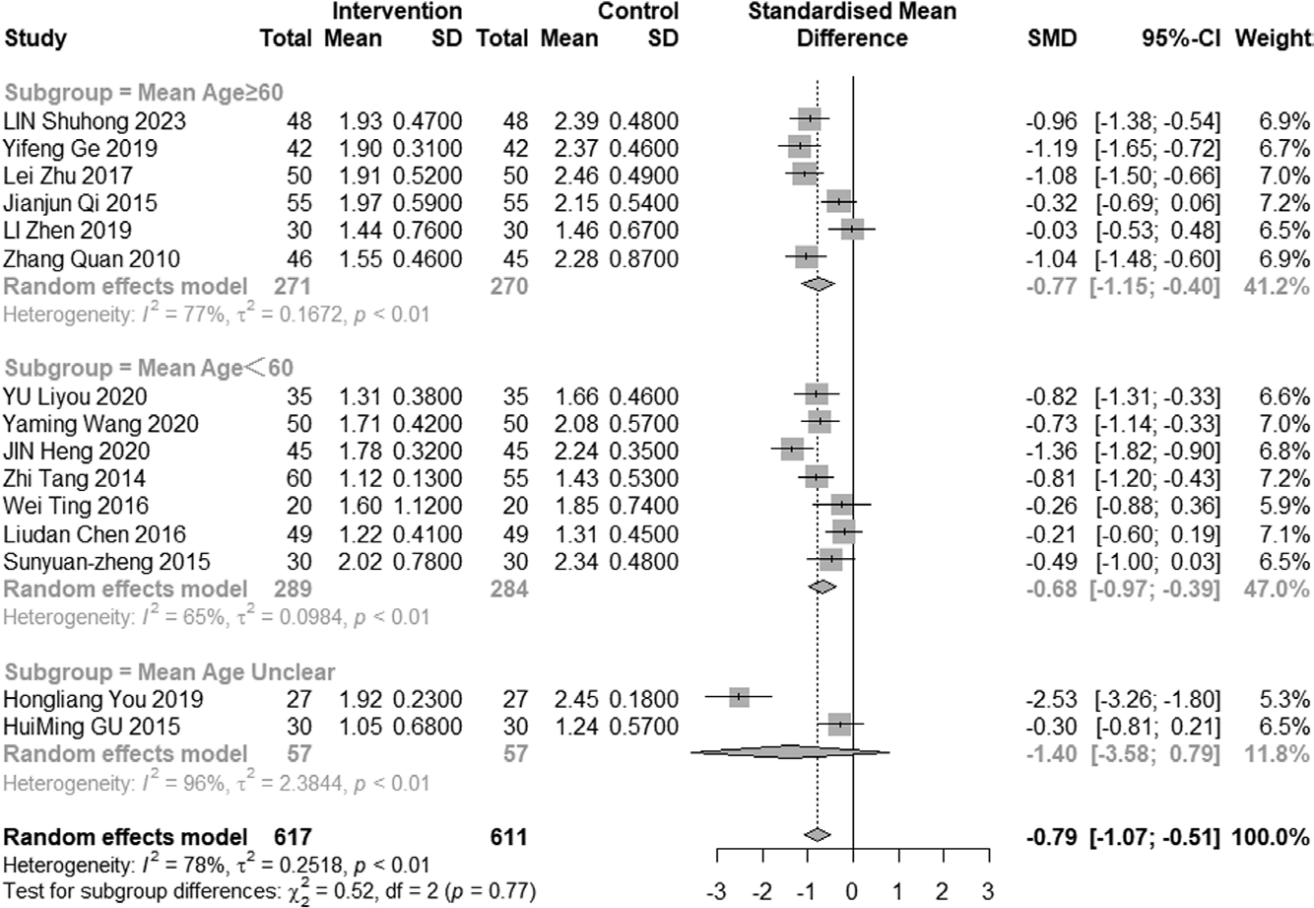
Subgroup analysis of TG according to mean age. TG = triglyceride.

**Figure 6.**
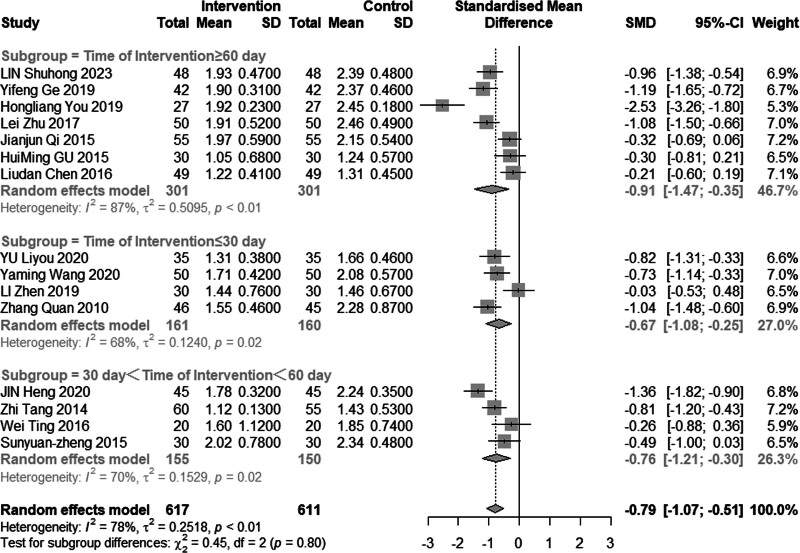
Subgroup analysis of TG according to intervention time. TG = triglyceride.

**Figure 7.**
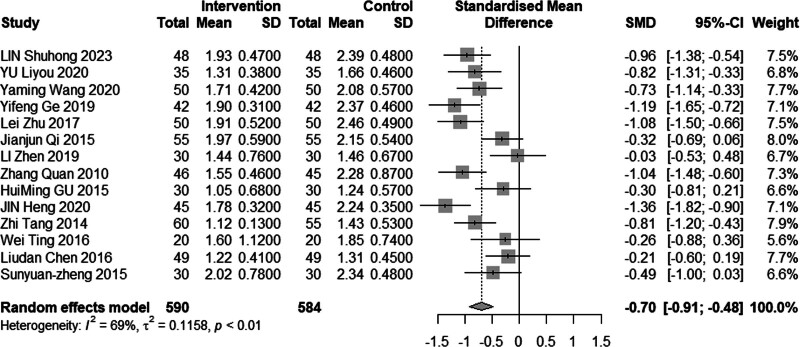
Sensitivity analysis of TG excluded JIN’s study. TG = triglyceride.

**Figure 8.**
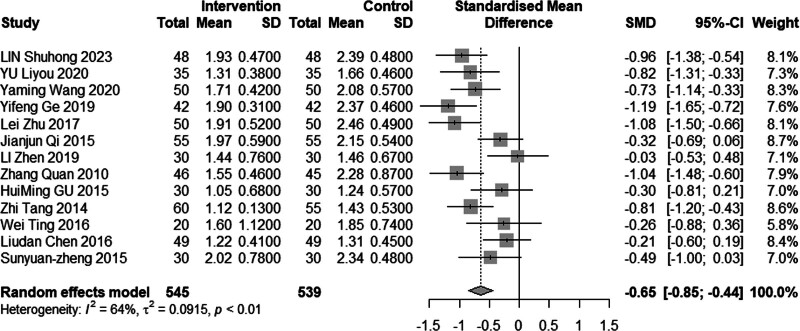
Sensitivity analysis of TG excluded JIN and Hong’s study. TG = triglyceride.

Fifteen studies (n = 1228) investigated the impact of intervention on TG levels. The results revealed significant heterogeneity (*I*^2^ = 78%, *P* < .01), leading us to employ a random effects model for analysis. The pooled MD was −0.79 mmol/L, with a 95% CI of −1.07 to −0.51, indicating that acupuncture combined with statin treatment effectively reduces TG levels, as depicted in Figure 4. Subgroup analysis results, illustrated in Figures 5 and 6, demonstrated a reduction in heterogeneity among individuals with a mean age less than 60 years or an average disease duration of less than or equal to 30 days. Notably, significant reduction in heterogeneity was observed upon excluding one or both of the studies by Heng et al^[14]^ and You et al^[21]^ (Figs. 7 and 8). Consequently, it is important to acknowledge that the heterogeneity may have originated from the inclusion of low-quality studies in the analysis.

#### 3.4.3. TC level

**Figure 9.**
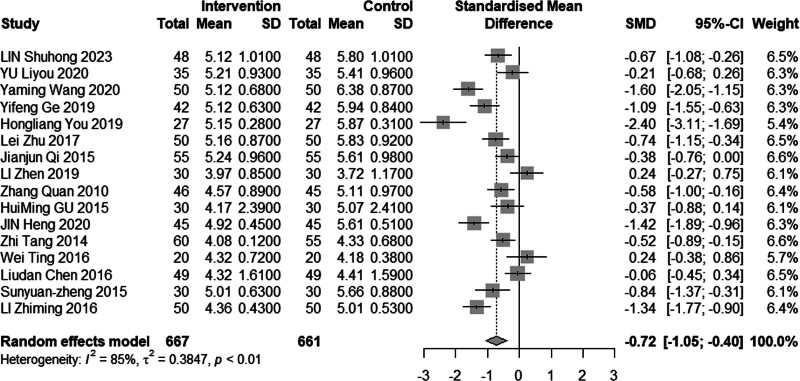
Meta analysis of TC level. TC = total cholesterol.

**Figure 10.**
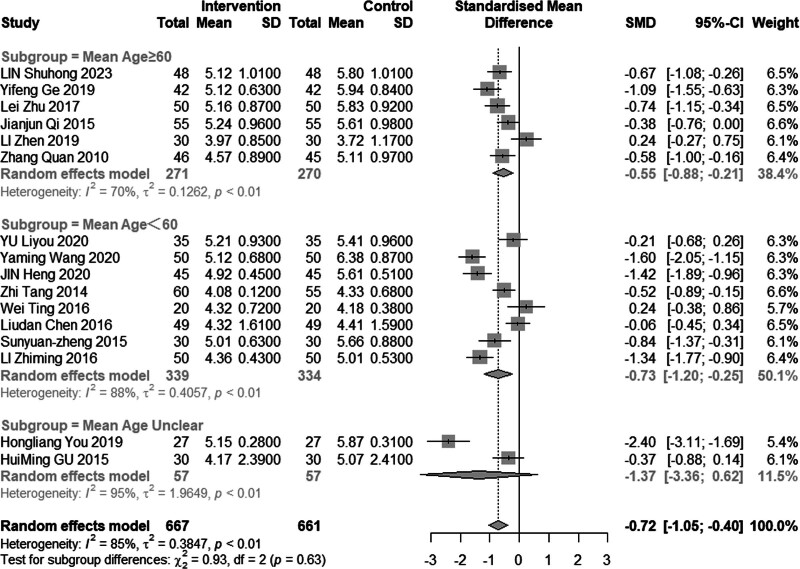
Subgroup analysis of TC according to mean age. TC = total cholesterol.

Sixteen studies (n = 1328) assessed TC level following intervention. The results exhibited significant heterogeneity (*I*^2^ = 85%, *P* < .01), prompting the utilization of a random effects model for analysis. The pooled MD was −0.72 mmol/L, with a 95% CI was −1.05 to −0.4, as depicted in Figure 9, indicating a moderate effect favoring acupuncture combined with statin therapy. Given the substantial heterogeneity in the results, we conducted subgroup analyses based on mean age, disease duration, and time of intervention, as well as sensitivity analysis involving one-by-one exclusion. As illustrated in Figure 10, the subgroup analysis revealed a reduction in heterogeneity only among patients aged 60 years or older (70% heterogeneity). However, no significant reduction in heterogeneity was observed in the one-by-one exclusion method.

#### 3.4.4. High-density lipoprotein cholesterol

**Figure 11.**
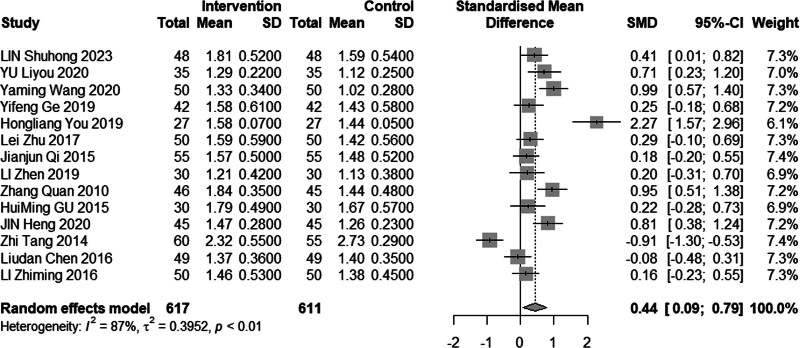
Meta analysis of HDL-C level. HDL-C = high-density lipoprotein cholesterol.

**Figure 12.**
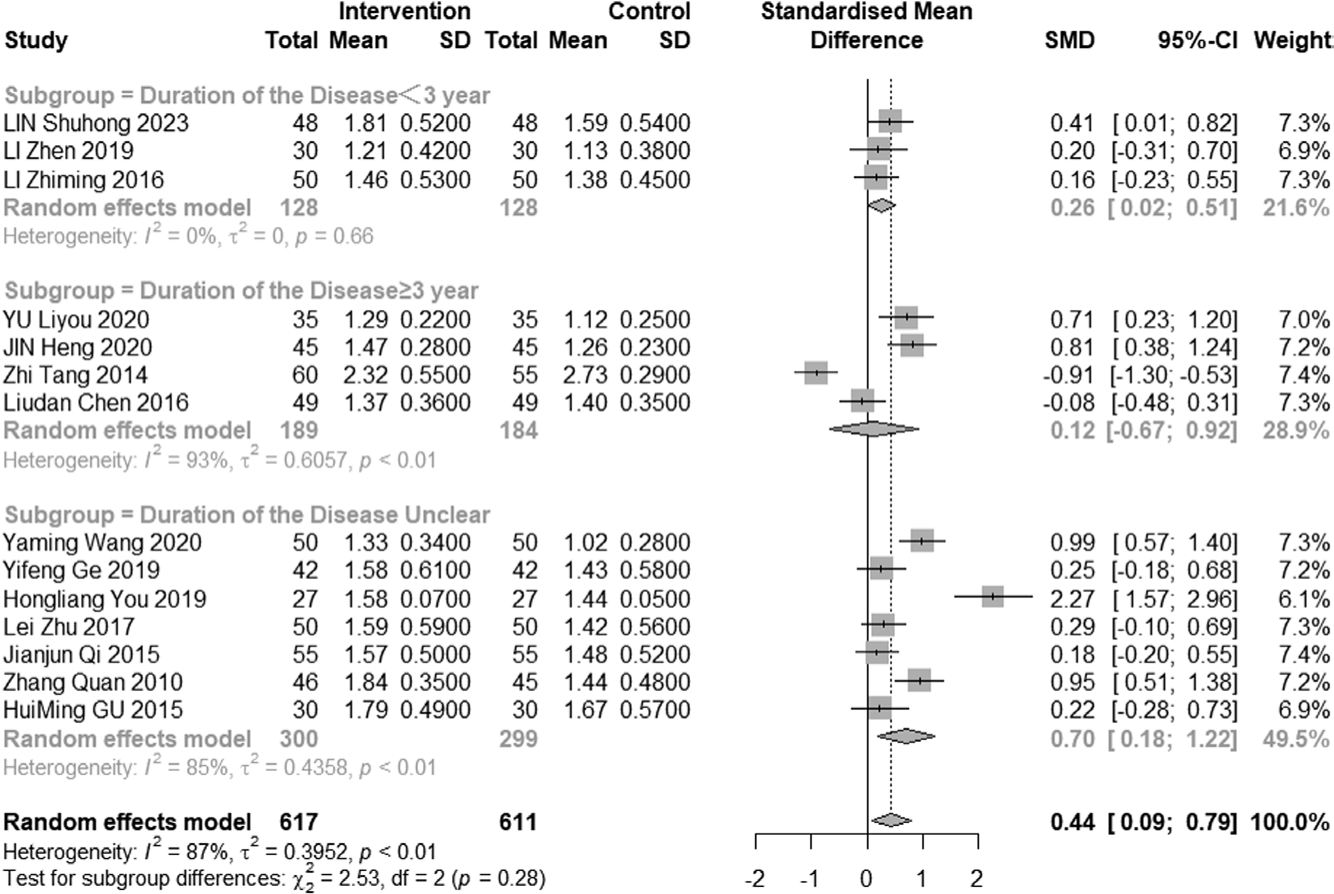
Subgroup analysis of HDL-C according to duration of the disease. HDL-C = high-density lipoprotein cholesterol.

**Figure 13.**
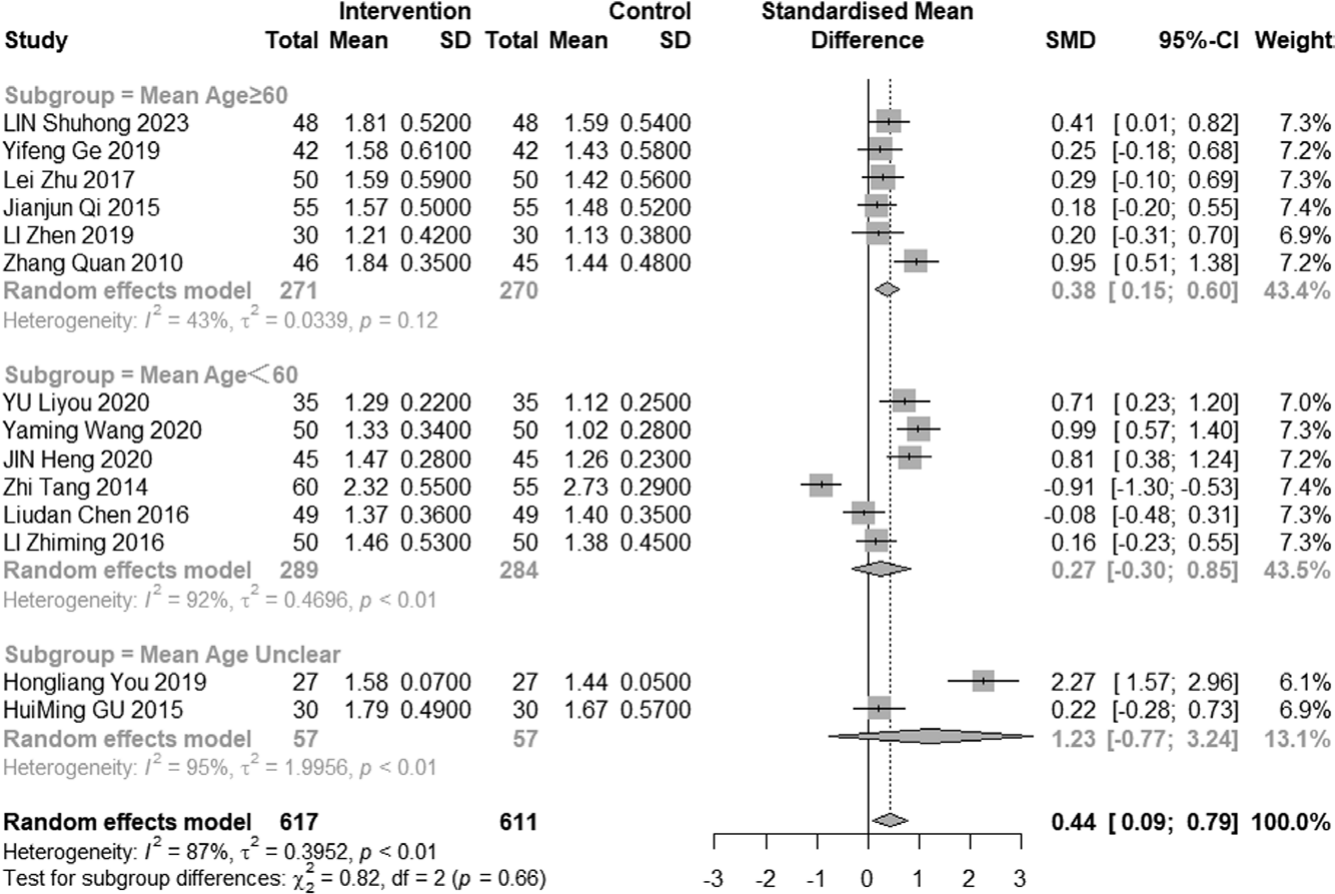
Subgroup analysis of HDL-C according to mean age. HDL-C = high-density lipoprotein cholesterol.

**Figure 14.**
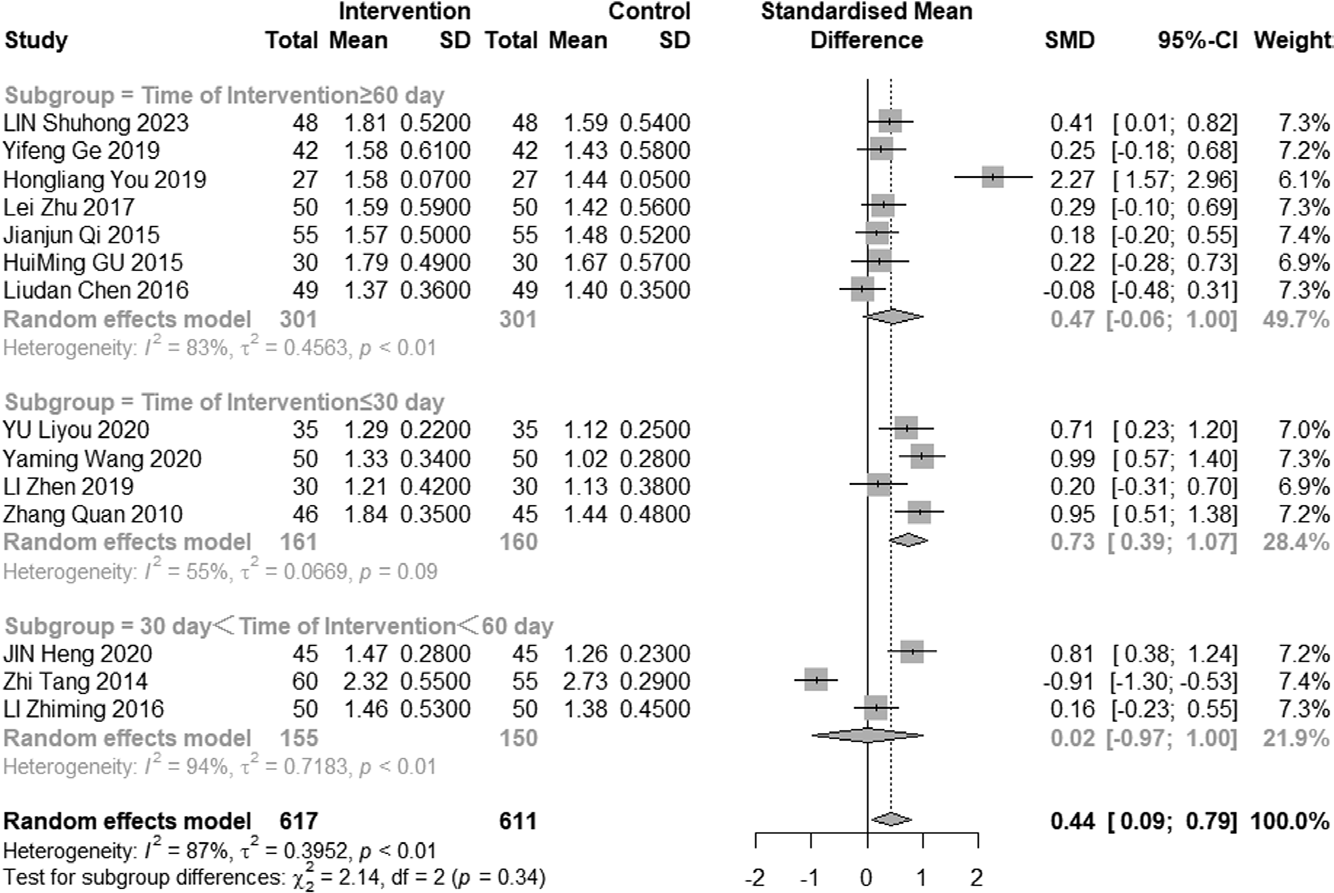
Subgroup analysis of HDL-C according to intervention time. HDL-C = high-density lipoprotein cholesterol.

**Figure 15.**
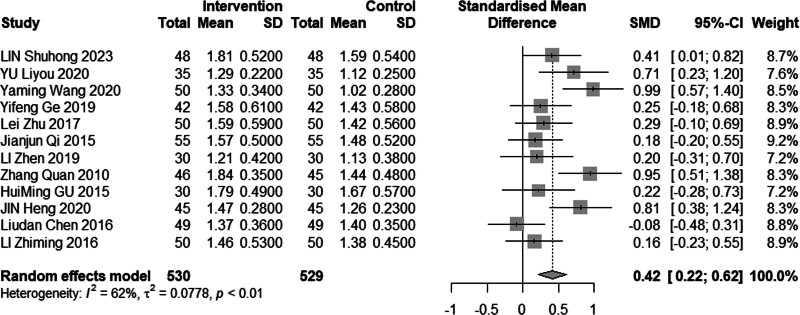
Sensitivity analysis of HDL-C excluded Hong and Zhi’s study. HDL-C = high-density lipoprotein cholesterol.

Eleven reviews (n = 1228) assessed the efficacy of combined acupuncture and statin therapy on HDL-C levels. The results exhibited significant heterogeneity (*I*^2^ = 87%, *P* < .01), prompting the utilization of a random effects model for analysis. The pooled MD was 0.44 mmol/L, with a 95% CI of 0.09 to 0.79, as illustrated in Figure 11. In the subgroup analysis (Figs. 12–14), heterogeneity was reduced to 0% in the group with a disease duration of less than 3 years, 43% in the group with an age of 60 years or older, and 55% in the group with a duration of intervention of less than or equal to 30 days. Upon excluding studies by You et al^[21]^ and Tang,^[15]^ the MD was 0.42 mmol/L, with a 95% CI of 0.08 to 0.22, and heterogeneity declined significantly to 64% (Fig. 15).

#### 3.4.5. Low-density lipoprotein cholesterol

**Figure 16.**
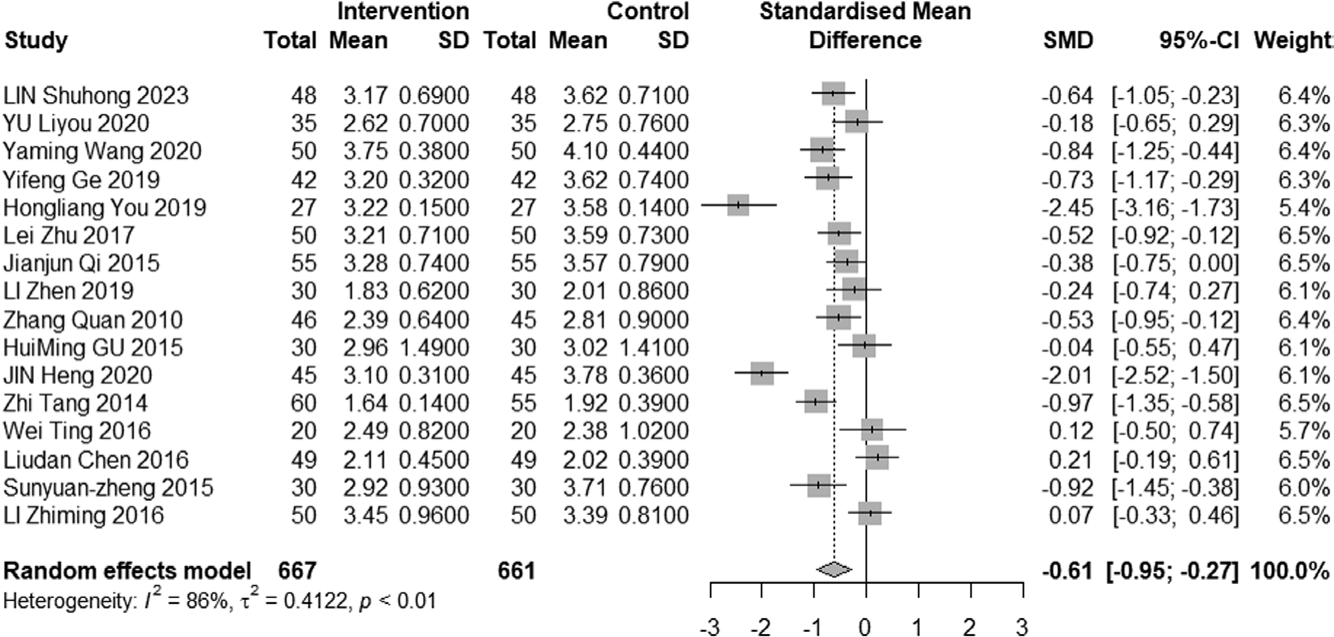
Meta analysis of LDL-C level. LDL-C = low-density lipoprotein cholesterol.

**Figure 17.**
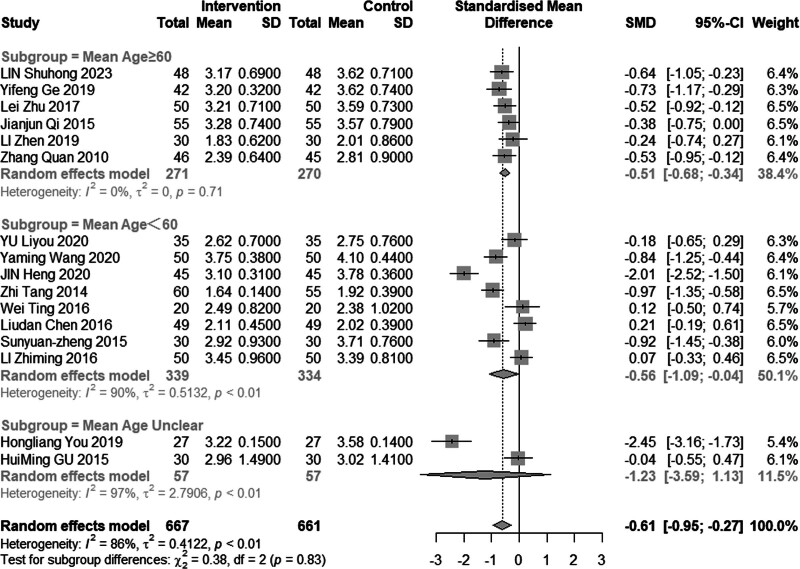
Subgroup analysis of LDL-C according to mean age. LDL-C = low-density lipoprotein cholesterol.

**Figure 18.**
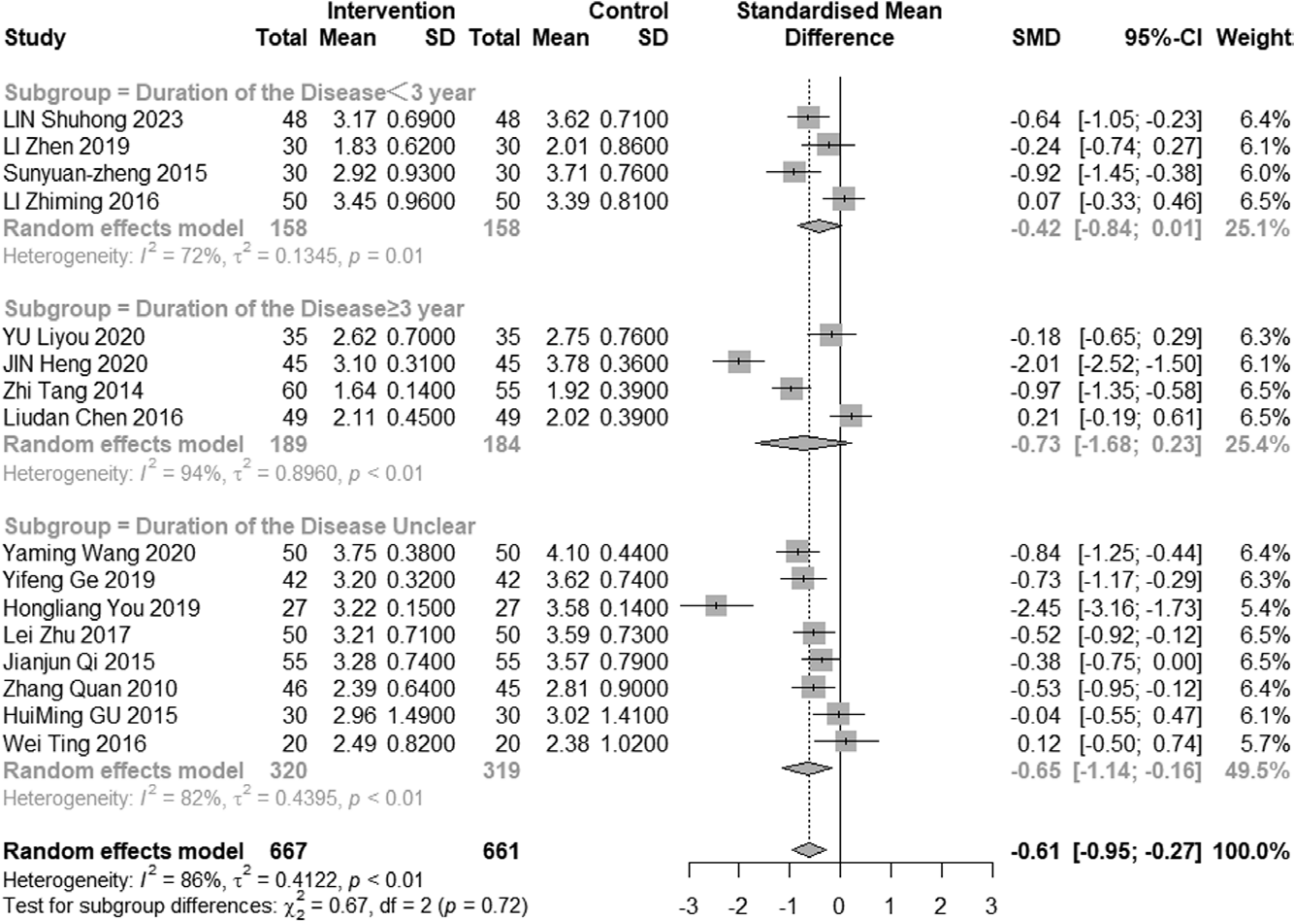
Subgroup analysis of LDL-C according to duration of the disease. LDL-C = low-density lipoprotein cholesterol.

**Figure 19.**
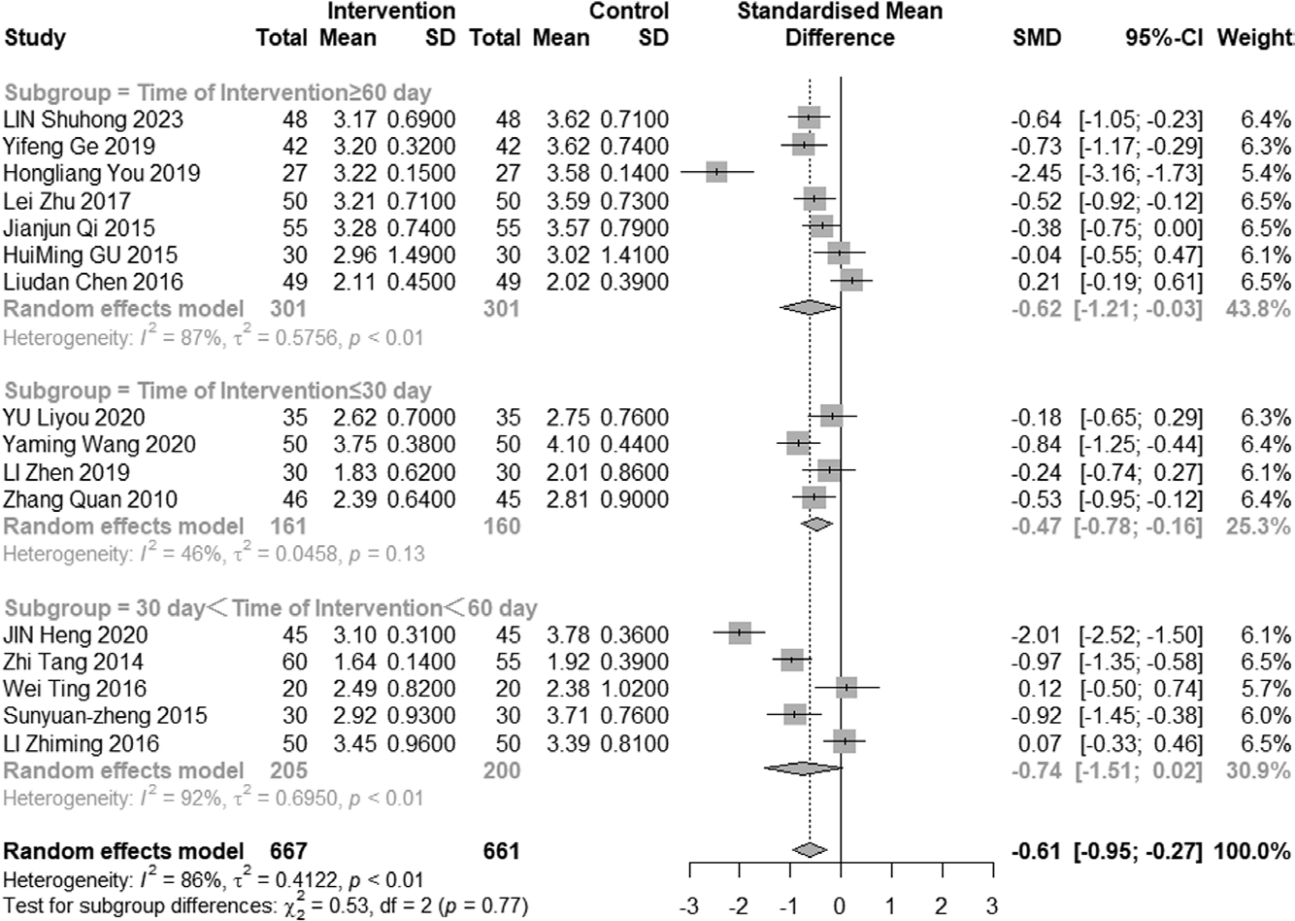
Subgroup analysis of LDL-C according to intervention time. LDL-C = low-density lipoprotein cholesterol.

**Figure 20.**
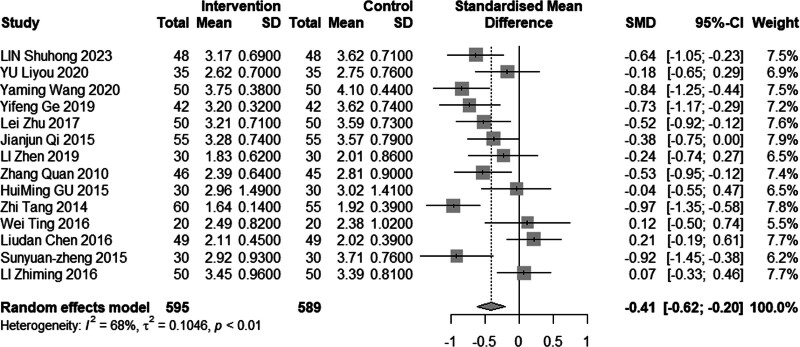
Sensitivity analysis of LDL-C excluded JIN and Hong’s study. LDL-C = low-density lipoprotein cholesterol.

Sixteen studies (n = 1328) investigated the impact of intervention on LDL-C levels. The results demonstrated significant heterogeneity (*I*^2^ = 86%, *P* < .01), leading to the use of a random effects model for analysis. The pooled MD was −0.61 mmol/L, with a 95% CI of −0.95 to −0.27, as depicted in Figure 16, indicating a favorable effect of acupuncture combined with statin treatment in reducing LDL-C levels. In the subgroup analysis, as illustrated in Figures 17 to 19, heterogeneity was reduced to 72% in the group with a disease duration of less than 3 years, 0% in the group with an age of 60 years or older, and 46% in the group with a duration of intervention of less than or equal to 30 days. Upon excluding studies by You et al^[21]^ and Heng et al,^[14]^ the MD was −0.41, with a 95% CI of −0.62 to −0.20, and the heterogeneity decreased to 68% (Fig. 20).

#### 3.4.6. Adverse reactions

In the included studies, a total of 7 articles provided data on adverse reaction outcomes, with the control group receiving statin intervention. Chen et al^[9]^ reported 4 cases (6.16%) of adverse reactions in the combined acupuncture group and 23 cases (46.93%) in the statin group. The incidence of adverse reactions in the combined acupuncture group was significantly lower than that in the statin group (*P* < .05). Wang et al^[19]^ reported 5 cases (10%) of adverse reactions in the combined acupuncture group and 3 cases (6%) in the statin group. Although the incidence of adverse reactions in the combined acupuncture group was lower than that in the statin group, the difference was not statistically significant (*P* > .05). Shuhong et al^[17]^ reported 1 case (2.08%) of adverse reaction in the combined acupuncture group and 8 cases (16.67%) in the statin group. The incidence of adverse reactions in the combined acupuncture group was significantly lower than that in the statin group (*P* = .036). The remaining 4 articles reported no adverse reactions in either the acupuncture combined group or the statin group alone. Therefore, based on the available evidence, it can be inferred that the rate of adverse reactions in the acupuncture combined with statin group is generally lower than that in the statin group alone.

#### 3.4.7. TCM syndrome integral

**Figure 21.**
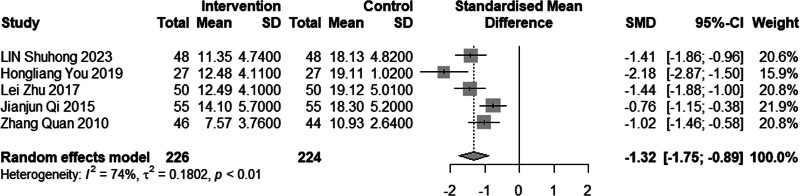
Meta analysis of TCM syndrome integral. TCM = traditional Chinese medicine.

Five studies comprising a total of 450 participants assessed the effectiveness of combined acupuncture and statin therapy for TCM syndrome integral. The pooled MD was −1.32, with a 95% CI of −1.75 to −0.89 (*P* < .01) (Fig. 21). These findings suggest that the combination of acupuncture and statin therapy in the management of dyslipidemia may lead to a further reduction in the TCM syndrome integral of patients, indicating a more favorable treatment outcome compared to statin monotherapy.

### 3.5. Publication bias

**Figure 22.**
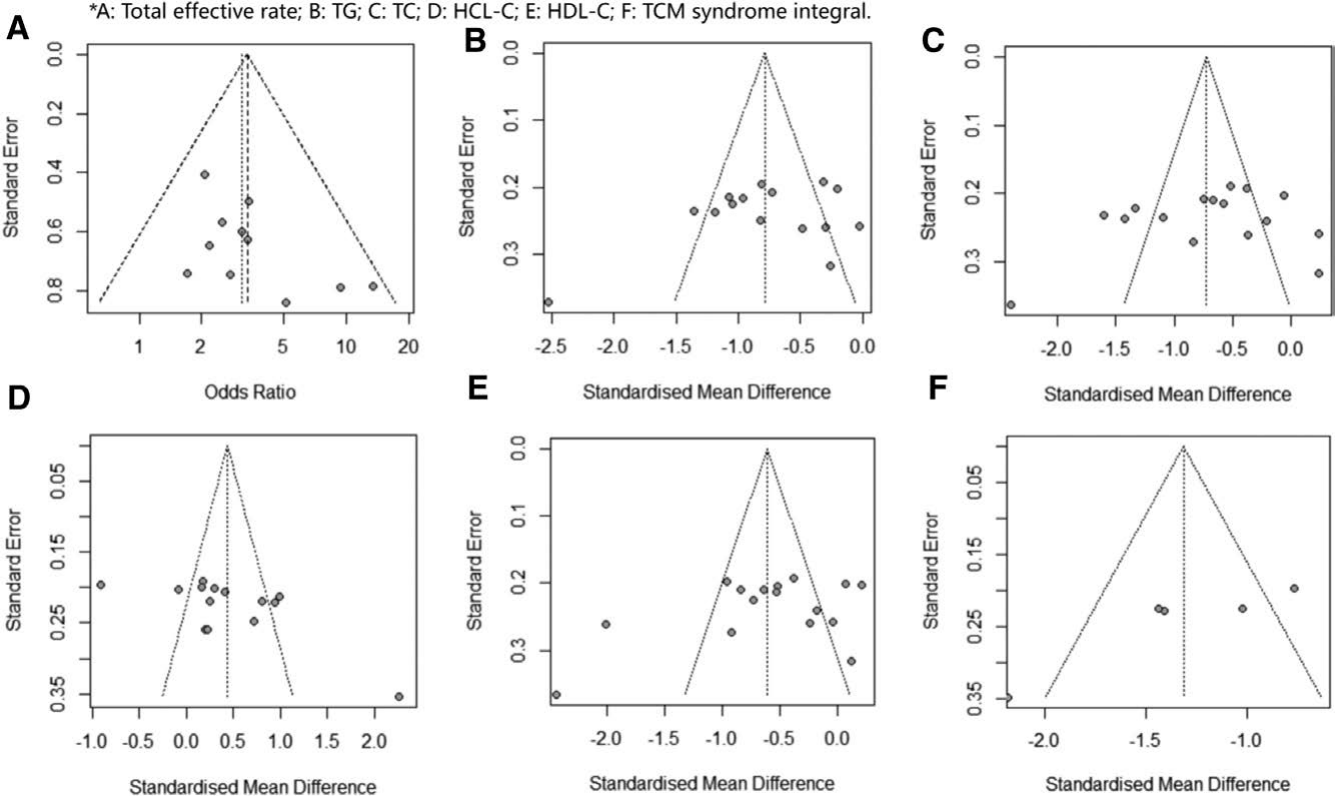
Publish funnel plots of bias.

We believe that there may be some subjectivity in the interpretation of Funnel plot (Fig. 22), so we conducted Begg test and Egger test on the above 6 outcome indicators on this basis. The operational results are shown in Table 2. The test results of all 6 outcome indicators are greater than 0.05. There was no suggested evidence of publication bias at post-intervention.

**Table 2:** Egger’s test and Begg’s test.

	Egger’s test	Result	Begg’s test	Result
Total effective rate	Prob > *z* = 0.069	No publication bias	Prob > *z* = 0.139	No publication bias
TG level	Prob > *z* = 0.291	No publication bias	Prob > *z* = 0.586	No publication bias
TC level	Prob > *z* = 0.415	No publication bias	Prob > *z* = 0.529	No publication bias
HDL-C level	Prob > *z* = 0.022	No publication bias	Prob > *z* = 0.043	No publication bias
LCL-C level	Prob > *z* = 0.163	No publication bias	Prob > *z* = 0.471	No publication bias

HDL-C = high-density lipoprotein cholesterol, LDL-C = low-density lipoprotein cholesterol, TC = total cholesterol, TG = triglyceride.

## 4. Discussion

Dyslipidemia is one of the important independent risk factors for disabling and fatal atherosclerotic diseases such as myocardial infarction and stroke.^[24]^ Prolonged inadequate control of serum lipids can lead to severe adverse outcomes. A mendelian randomization study,^[25]^ featured in the New England Journal of Medicine, revealed that for every 10 milligrams per deciliter reduction in LDL-C level, the risk of cardiovascular events diminished by 17.6% (ACLY score) or 16.4% (HMGCR score). Additionally, a large-scale prospective clinical meta-analysis demonstrated a substantial reduction in ASCVD risk with the decrease of non-high-density lipoprotein cholesterol.^[26]^ Data from 6 community-based cohorts^[27]^ exhibited an inverse and linear correlation between HDL-C and coronary heart disease risk up to a threshold of ≤90 mg/dL. A comprehensive 19-year Chinese cohort study revealed a notable increase in ASCVD risk with rising TG levels, with serum TG level independently and positively linked to ASCVD risk.^[28]^ Effective dyslipidemia management can lead to reduced hospitalization rates, lower fatality rates, and improved survival rates.^[24]^

However, the use of statins as the primary medication for lipid reduction has limitations. A meta-analysis^[29]^ revealed that long-term statin use resulted in only a 4% to 10% increase in HDL-C. High-intensity rosuvastatin (20 mg/d) was found to increase HDL-C by only 6.1%.^[30]^ Moreover, research by Preiss et al^[31]^ indicated that intensive statin treatment significantly raised the risk of new-onset diabetes. Statins have limited effects in reducing TG, and their combination with fibrates may increase the risk of liver damage, myositis, and myopathy due to similar metabolic pathways. Furthermore, statin intolerance also poses challenges for some dyslipidemia patients and medical staff.^[32]^

Our study demonstrates that compared to using statin alone, the combined use of acupuncture and statin yields a significant therapeutic effect (total effective rate odds ratio value = 3.12, 95% CI = 2.17 to 4.48). The further reduction of TC (MD = −0.72 mmol/L, 95% CI = −1.05 to −0.4) and LDL-C (MD = −0.61 mmol/L, 95% CI = −0.95 to −0.27) at moderate statin doses may offer a new treatment option for statin intolerance, uncontrolled lipid levels, and the risk of new-onset diabetes mellitus associated with high-intensity statin therapy. Simultaneously, it effectively improves HD-C and TG levels (MD = −0.36 mmol/L, 95% CI = −0.61 to −0.11). The combination therapy also exhibited a favorable safety profile, with a lower incidence of adverse reactions compared to using statin alone. This indicates that acupuncture combined with statin is a safer approach for dyslipidemia treatment.

However, our study also acknowledges certain limitations. The heterogeneity across the included studies was high, likely due to differences in patient characteristics, treatment regimens, and study methodologies. Additionally, the sample size of the studies was relatively small, warranting the need for larger, multi-center randomized controlled trials in the future. Standardized research and testing methods, as well as the establishment of effective evaluation systems, are essential to improve the quality and reliability of findings in this field. Furthermore, the mechanism of action of acupuncture in treating dyslipidemia is not fully understood, and the existing efficacy evaluation systems may not fully capture the benefits of TCM treatments. Future research should focus on elucidating the mechanisms of acupuncture and combined treatments, as well as developing a unified evaluation system for the therapeutic effects of acupuncture and moxibustion on dyslipidemia.

In conclusion, while statins remain a cornerstone of dyslipidemia management, the potential of acupuncture and moxibustion therapy as a complementary and alternative treatment warrants further investigation. Larger clinical trials and mechanistic studies are needed to fully explore the potential of this approach and establish its place in the management of dyslipidemia. The development of standardized research and testing methods, as well as effective evaluation systems, is crucial to improving the quality and reliability of findings in this field.

## Author contributions

**Data curation:** Xinyu Liu, Kun Chen.

**Formal analysis:** Xinyu Liu, Kun Chen, Fujian Chen.

**Methodology:** Xinyu Liu, Fujian Chen.

**Software:** Xinyu Liu.

**Writing – original draft:** Xinyu Liu, Kun Chen, Fujian Chen.

**Writing – review & editing:** Fujian Chen.

## References

[1] Sandesara PB Virani SS Fazio S Shapiro MD. The forgotten lipids: triglycerides, remnant cholesterol, and atherosclerotic cardiovascular disease risk. Endocr Rev. 2019;40:537–57. 30312399 10.1210/er.2018-00184 PMC6416708

[2] Yamashita K. [Induction of experimental protoporphyria in hairless mice griseofulvin – strain differences in murine protoporphyria]. Nihon Hifuka Gakkai zasshi Japanese J Dermatol. 1988;98:677–81. 3249408

[3] Liu T Zhao D Qi Y. Global trends in the epidemiology and management of dyslipidemia. J Clin Med. 2022;11:6377. 36362605 10.3390/jcm11216377 PMC9656679

[4] Zengwu W Jing L Jianjun L. Chinese guidelines for lipid management. Chin Circulation J. 2023;38:237–71.

[5] Xiaoyu Z. Guiding Principles for Clinical Research of New Chinese Medicine Drugs Trial Implementation. China Medical Science and Technology Press; 2002:402.

[6] Cumpston M Li T Page MJ. Updated guidance for trusted systematic reviews: a new edition of the Cochrane Handbook for Systematic Reviews of Interventions. Cochrane Database Syst Rev. 2019;10:ED000142. 31643080 10.1002/14651858.ED000142 PMC10284251

[7] Page MJ McKenzie JE Bossuyt PM. The PRISMA 2020 statement: an updated guideline for reporting systematic reviews. BMJ. 2021;372:n71. 33782057 10.1136/bmj.n71 PMC8005924

[8] Zhiming L Meiying L Changjun H ShuSsheng L Yuqin X Jianhua M. Clinical observation of acupuncture in the treatment of hypercholesterolemia. Chin Med Modern Distance Educ China. 2016;14:106–8.

[9] Chen L Yi W Tao Y. Effect and safety of acupuncture on blood lipids in obesity and hyperlipidemia. J Pract Tradit Chin Med. 2016;32:464–5.

[10] Quan Z Liping Z Rong B editors. Acupuncture in the Treatment of Elderly Patients with Hyperlipidemia (Blood Stasis) in Clinical Research. The 3rd International Conference on Science and Technology for the Modernization of Traditional Chinese Medicine. Chinese Association of Traditional Chinese Medicine; 2010.

[11] Ting W. Observation on therapeutic effect of acupuncture and xinmaitong capsule in treating hyperlipidemia combined with western medicine. J Zhejiang Chin Med Univ. 2016;40:63–5.

[12] Gu H. Spleen-strengthening acupuncture intervention in hyperlipidemia was clinically observed in 30 cases. Yunnan J Tradit Chin Med Materia Medica. 2015;36:50–1.

[13] Zhen L Hong-yue N. Effect of deep needling with elongate needles on blood lipid levels in patients with post-stroke dyspepsia coupled with hyperlipidemia. Shanghai J Acu-mox. 2019;38:40–4.

[14] Heng J Xue-song L Qiong W Hong-xing Z. Efficacy of acupuncture for hyperlipidemia and its effects on blood lipids. Shanghai J Acupunct Moxibustion. 2020;39:1215–9.

[15] Tang Z. Observation on the efficacy and safety of electroacupuncture stimulation of Yinlingquan combined with atorvastatin in the treatment of hyperlipidemia. Inner Mongolia J Tradit Chin. 2014;33:56–7.

[16] Sun YZ Song J. Clinical trials for treatment of primary hyperlipidemia by using acupuncture in combination with Lipitor. Acupunct Res. 2015;40:61–4. 25845223

[17] Shuhong L Jiayuan C. Clinical effect of simvastatin combined with warm acupuncture and Chinese herbal tea in the treatment of coronary heart disease with hyperlipidemia. Chin J Clin Rational Drug Use. 2023;16:5–7 + 11.

[18] Liyou Y Zhidong W Zhangiang L Xueying L Yanjun W. Clinical observation of Hewei Huatan Jiangzhuo decoction combined with acupuncture in the treatment of hyper-lipidemia with phlegm-turbid stagnation type. Hebei J TCM. 2020;42:202–5 + 9.

[19] Wang Y Xiao K Liu X Chen Q. Study on the efficacy of warm acupuncture and aconite Center-Rectifying Decoction combined with Western medicine in the treatment of spleen and kidney yang deficiency dyslipidemia. Shaanxi J Tradit Chin Med. 2020;41:770–3.

[20] Ge Y. To observe the efficacy of warm acupuncture and traditional Chinese herbal tea combined with simvastatin in the treatment of patients with hyperlipidemia of coronary atherosclerotic heart disease. Cardiovasc Disease Electronic J Integrated Tradit Chin Western Med. 2019;7:161.

[21] You H. Clinical study of warm acupuncture and Chinese herbal tea combined with simvastatin in the treatment of coronary heart disease complicated with hyperlipidemia. J Must-Read For Health. 2019;22:129–30.

[22] Zhu L. Clinical study of warm acupuncture and Chinese herbal tea combined with simvastatin in the treatment of coronary heart disease complicated with hyperlipidemia. Asia Pacific Tradit Med. 2017;13:112–4.

[23] Qi J Ji Y Liu S Zeng X Zhao G. Warm acupuncture and Chinese herbal tea combined with simvastatin in the treatment of coronary heart disease hyperlipidemia in 55 cases. China Pharmaceuticals. 2015;24:81–3.

[24] Lu H. Chinese expert consensus on integrated lipid management in HIV/AIDS. Zhonghua nei ke za zhi. 2023;62:661–72. 37263949 10.3760/cma.j.cn112138-20230321-00165

[25] Ference BA Ray KK Catapano AL. Mendelian randomization study of ACLY and cardiovascular disease. N Engl J Med. 2019;380:1033–42. 30865797 10.1056/NEJMoa1806747 PMC7612927

[26] Ference BA Ginsberg HN Graham I. Low-density lipoproteins cause atherosclerotic cardiovascular disease. 1. Evidence from genetic, epidemiologic, and clinical studies. A consensus statement from the European Atherosclerosis Society Consensus Panel. Eur Heart J. 2017;38:2459–72. 28444290 10.1093/eurheartj/ehx144 PMC5837225

[27] Wilkins JT Ning H Stone NJ. Coronary heart disease risks associated with high levels of HDL cholesterol. J Am Heart Assoc. 2014;3:e000519. 24627418 10.1161/JAHA.113.000519 PMC4187512

[28] Huanhuan L Yong W Ying L. Relationship between serum triglyceride and the risk of atherosclerotic cardiovascular disease: a prospective study. Chin Circ J. 2019;34:122–7.

[29] McTaggart F Jones P. Effects of statins on high-density lipoproteins: a potential contribution to cardiovascular benefit. Cardiovasc Drugs Ther. 2008;22:321–38. 18553127 10.1007/s10557-008-6113-z PMC2493531

[30] Mora S Glynn RJ Ridker PM. High-density lipoprotein cholesterol, size, particle number, and residual vascular risk after potent statin therapy. Circulation. 2013;128:1189–97. 24002795 10.1161/CIRCULATIONAHA.113.002671 PMC3807967

[31] Sattar N Preiss D Murray HM. Statins and risk of incident diabetes: a collaborative meta-analysis of randomised statin trials. Lancet. 2010;375:735–42. 20167359 10.1016/S0140-6736(09)61965-6

[32] Serban MC Colantonio LD Manthripragada AD. Statin intolerance and risk of coronary heart events and all-cause mortality following myocardial infarction. J Am Coll Cardiol. 2017;69:1386–95. 28302290 10.1016/j.jacc.2016.12.036

